# Indoxyl Sulphate is Associated with Atrial Fibrillation Recurrence after Catheter Ablation

**DOI:** 10.1038/s41598-018-35226-5

**Published:** 2018-11-22

**Authors:** Fumi Yamagami, Kazuko Tajiri, Kosuke Doki, Masayuki Hattori, Junya Honda, Satoshi Aita, Tomohiko Harunari, Hiro Yamasaki, Nobuyuki Murakoshi, Yukio Sekiguchi, Masato Homma, Naohiko Takahashi, Kazutaka Aonuma, Akihiko Nogami, Masaki Ieda

**Affiliations:** 1 Department of Cardiology, Faculty of Medicine, University of Tsukuba, Tsukuba, Japan; 2 Department of Pharmaceutical Sciences, Faculty of Medicine, University of Tsukuba, Tsukuba, Japan; 3 Department of Cardiology and Clinical Examination, Oita University Faculty of Medicine, Oita, Japan

## Abstract

Renal dysfunction results in the accumulation of various uremic toxins, including indoxyl sulphate (IS), and is a major risk factor for atrial fibrillation (AF). Experimental studies have demonstrated that IS exacerbates atrial remodelling via oxidative stress, inflammation, and fibrosis. However, its clinical impact on AF-promoting cardiac remodelling has not been described. Therefore, the purpose of this study was to clarify the relationship between basal IS levels and the 1-year outcomes after catheter ablation for the treatment of AF. Our prospective observational study included data from 125 patients with AF who underwent catheter ablation. Over a 1-year follow-up period, AF recurrence was identified in 21 patients. The 1-year AF-free survival was significantly lower in patients with high serum IS levels (≥0.65 μg/mL) than in those with low IS levels (60.1 ± 10.4% *versus* 85.2 ± 3.9%, *P* = 0.007). Univariable analysis identified that an IS concentration ≥ 0.65 μg/mL was associated with AF recurrence (hazard ratio [HR] = 3.10 [1.26–7.32], *P* = 0.015), and this association was maintained in multivariate analysis (HR = 3.67 [1.13–11.7], *P* = 0.031). Thus, in patients undergoing AF ablation, serum IS levels at baseline independently predict the recurrence of arrhythmia.

## Introduction

Several clinical trials have reported a close association between atrial fibrillation (AF) and chronic kidney disease (CKD)^1–5^. For example, the burden of AF is increased among patients with CKD, and AF itself increases as a function of CKD severity. The relationship between renal function and AF recurrence after catheter ablation for AF was addressed in recent reports^6–8^. Patients with a lower estimated glomerular filtration rate (eGFR) at baseline had a greater risk of recurrence after radiofrequency catheter ablation for AF. Atrial remodelling due to CKD was thought to be responsible for the poor outcome after AF ablation in patients with CKD; however, the exact mechanism of this association remains to be elucidated.

Indoxyl sulphate (IS) is among the most representative uremic toxins derived from the metabolism of dietary protein by the gut microbiota and has been implicated in the pathogenesis of various cardiovascular diseases^9–11^, including AF^12^. Experimental studies in animal models have shown that IS can exacerbate AF via its effect on cardiac fibrosis and inflammation, with enhanced oxidative stress and reduced anti-oxidative defense^13,14^. These effects have been shown to induce arrhythmogenesis in pulmonary veins, sinoatrial nodes, and atria isolated from rabbit hearts^15^. Recently, Aoki *et al*.^16^ demonstrated, in a rat model of CKD, that IS increases the inducibility of AF, and that circulating levels of IS can be significantly attenuated by using an absorbent of uremic toxins, such as AST-120. Thus, it is plausible that IS may contribute to atrial remodelling, although there is limited clinical evidence in support of the association between IS and AF.

Therefore, the aim of our study was to investigate the relationship between IS and recurrent AF in patients after radiofrequency catheter ablation (RFCA) for AF.

## Methods

### Study population

This prospective observational study was conducted at the University of Tsukuba Hospital, Tsukuba, Japan, between April 2009 and March 2011. We enrolled 125 consecutive patients with non-valvular AF scheduled for RFCA for AF. Patients who had hemodynamically significant (moderate-to-severe) valvular disease, thrombus in the left atrium (LA), uncontrolled thyroid dysfunction, pre-procedural significant coronary artery stenosis, a previous myocardial infarction or cardiac surgery in the previous 3 months, contraindications to anticoagulant therapy, stage 4 or 5 CKD, or pregnancy were excluded.

All enrolled patients had symptomatic paroxysmal or persistent AF and had not responded to treatment with one or more antiarrhythmic drugs. Long-standing persistent AF was defined as any AF episode lasting for longer than 1 year. A detailed medical history regarding AF and related cardiovascular and/or systemic conditions was obtained from all patients. The CHADS_2_ score was calculated for each patient based on a point system, as previously described^17^. Transthoracic echocardiography was also performed to assess left ventricular function and the left atrial volume index (LAVI) before RFCA.

The study was in compliance with the principles outlined in the Declaration of Helsinki and was approved by Ethics Committee, University of Tsukuba Hospital. Before ablation, written informed consent was obtained from all the patients.

### Study endpoint

The endpoint of this study was recurrence of AF or atrial tachycardia after a blanking period of 3 months.

### Serum IS concentration

Blood samples were collected from patients just before ablation and serum was stored at −80 °C. Serum levels of IS and indole-3 acetic acid (IAA) were measured by reversed-phase high-performance liquid chromatography using a conventional octadecylsilyl silica column and a fluorescence detector, according to a previously described method, with minor modifications^18^. The coefficients of variation for intra-day and inter-day assays were <3%.

### Classification of CKD

Classification of the CKD stage was determined using the eGFR, calculated at baseline. The eGFR was calculated using the estimation equation for Japanese patients with CKD^19^. This equation calculates the eGFR from serum creatinine, adjusted for age and sex, using the following formula: (eGFR [mL/min/1.73 m^2^] = 194 × age^−0.287^ × serum creatinine^−1.094^ × [0.739 for women]). The study population was divided into three subgroups: patients with an eGFR ≥ 90 mL/min/1.73 m^2^ (CKD stage 1), patients with an eGFR between 60 and 89.9 mL/min/1.73 m^2^ (CKD stage 2), and patients with an eGFR between 30 and 59.9 mL/min/1.73 m^2^ (CKD stage 3). The creatinine clearance (CrCl) was evaluated using the Cockcroft–Gault formula: CrCl (mL/min) = ([140 - age] × body weight [kg] × [0.85 if female])/(72 × serum creatinine [mg/dL]).

### Catheter ablation

Procedures were performed under general anaesthesia, aiming at an anticoagulation time of 300 s. An irrigated-tip catheter was used to deliver radiofrequency energy in all patients. A circular lasso catheter was used to confirm the pulmonary vein (PV) and to map the LA area. After trans-septal puncture, PV isolation (PVI) was performed. After PVI, a bidirectional block was systematically obtained in all veins. In the case of persistent AF, complete electrical isolation of the PVs was confirmed after restoration of a sinus rhythm. If the AF was sustained or recurred despite the internal cardioversion after PVI, a stepwise approach was used, including linear ablation of the LA roof, superior vena cava isolation, and/or ablation of complex fractionated atrial electrograms.

### Follow-up

Follow-up was performed at 1, 3, 6, and 12 months, or earlier if a patient developed symptoms consistent with recurrent AF. The average follow-up period was 11.7 months (range, 3.2 to 12 months). Recurrence was defined as any evidence of an episode of atrial arrhythmia lasting for more than 30 s after a 3-month blanking period.

### Statistical analysis

Analyses were performed using JMP software (version 10). Values are reported as the mean ± standard deviation (SD) for continuous variables, and proportions for categorical variables. The optimal cut-off point was determined by the maximum Youden index based on receiver operating characteristic (ROC) curve analysis, and areas under the ROC curve (AUCs) were calculated. The association between serum levels of IS or IAA and other factors was evaluated using Pearson’s correlation coefficients. Differences between continuous values were assessed using an unpaired, two-tailed, *t*-test for normally distributed continuous variables, and a Mann–Whitney test for skewed variables. The chi-square statistic was used for testing relationships between categorical variables. The association between baseline variables and AF recurrence was evaluated using univariate and multivariate Cox proportional hazards analysis. AF-free survival curves were analysed according to the Kaplan–Meier method and compared by the log-rank test. A *P*-value < 0.05 was considered significant.

## Results

### Study population

Of the 125 consecutive patients with AF enrolled, 20 were excluded from the analysis due to missing pre-echocardiographic data (5 patients) and because of a lack of follow-up (15 patients). Relevant data of the remaining 105 patients (83.8% men, mean age: 60.0 ± 10.8 years) included in the analysis are reported in Table 1. Over the 1-year follow-up after ablation, 21 (20.0%) patients developed AF recurrence (AF in 16, uncommon atrial flutter in 1, and atrial tachycardia in 4 patients). There were no significant differences in baseline IS and IAA levels between patients who developed AF-recurrence and those who did not (IS, 0.51 ± 0.33 *vs* 0.43 ± 0.31 μg/mL, *P* = 0.27; IAA, 0.14 ± 0.05 *vs* 0.16 ± 0.10 μg/mL, *P* = 0.31, respectively). After calculating the maximum Youden index (sensitivity + specificity − 1) and the AUC, the optimal cut-off values of serum levels of IS and IAA were found to be 0.65 μg/mL and 0.17 μg/mL, respectively (Table 2).

**Table 1:** Baseline characteristics.

	All (n = 105)	IS ≥ 0.65 μg/mL (n = 23)	IS < 0.65 μg/mL (n = 82)	*P*-value
Age (years)	60.0 ± 10.8	62.9 ± 11.3	59.2 ± 10.6	0.14
Male sex	88 (83.8)	20 (87.0)	68 (82.9)	0.64
Body mass index (kg/m^2^)	23.6 ± 2.9	23.7 ± 3.2	23.6 ± 2.9	0.94
Paroxysmal AF	69 (65.7)	14 (60.9)	55 (67.1)	0.58
Long-standing AF	19 (19.1)	7 (30.4)	12 (14.6)	0.082
Duration of AF history (years)	5.5 ± 5.7	8.4 ± 6.9	4.8 ± 5.2	0.010
**Medication**				
ACEIs/ARBs	44 (41.9)	10 (43.5)	34 (41.5)	0.86
Statins	33 (31.4)	5 (21.7)	28 (34.2)	0.26
Beta-blockers	58 (55.2)	19 (82.6)	39 (47.6)	0.003
Class I AADs	59 (56.2)	12 (52.2)	47 (57.3)	0.66
Class III AADs	34 (32.4)	10 (43.5)	24 (29.3)	0.20
Class IV AADs	16 (15.2)	2 (8.7)	14 (17.1)	0.32
Hypertension	52 (49.5)	13 (56.5)	39 (47.6)	0.45
Diabetes mellitus	14 (13.3)	3 (13.0)	11 (13.4)	0.96
Dyslipidaemia	48 (45.7)	9 (39.1)	39 (47.6)	0.47
CHADS_2_ score	0.81 ± 0.84	1.04 ± 0.88	0.74 ± 0.83	0.13
**Echocardiogram parameters**				
LVEF (%)	65.2 ± 11.2	66.3 ± 12.4	64.9 ± 10.9	0.62
LAVI (mL/m^2^)	36.3 ± 18.3	44.7 ± 22.0	33.8 ± 16.4	0.012
eGFR (mL/min/1.73 m^2^)	76.1 ± 18.5	68.2 ± 20.9	78.4 ± 17.3	0.019
**CKD stage**				0.021
Stage 1	19 (18.1)	4 (17.4)	15 (18.3)	
Stage 2	69 (65.7)	11 (47.8)	58 (70.7)	
Stage 3	17 (16.2)	8 (34.8)	9 (11.0)	
BNP (pg/mL)	78.9 ± 91.6	114.1 ± 111.7	69.5 ± 83.8	0.042
CRP (mg/mL)	0.097 ± 0.102	0.114 ± 0.111	0.092 ± 0.099	0.34
IS (μg/mL)	0.45 ± 0.31	0.93 ± 0.25	0.31 ± 0.15	< 0.001
IAA (μg/mL)	0.16 ± 0.10	0.18 ± 0.07	0.15 ± 0.10	0.097

Values are given as mean ± SD or number (%).

AAD, anti-arrhythmic drug; AF, atrial fibrillation; ACEI, angiotensin-converting enzyme inhibitor; ARB, angiotensin receptor antagonist; BNP, B-type natriuretic peptide; CRP, C-reactive protein; eGFR, estimated glomerular filtration rate; IAA, indole-3 acetic acid; IS, indoxyl sulphate; LAVI, left atrial volume index; LVEF, left ventricular ejection fraction.

**Table 2:** Optimal cut-off point based on AF recurrence.

	Median value	Range	AUC	Cut-off value
IS (μg/mL)	0.36	0–1.74	0.576	0.65
IAA (μg/mL)	0.13	0.04–0.56	0.522	0.17

AF, atrial fibrillation; AUC; area under the curve, IAA, indole-3 acetic acid; IS, indoxyl sulphate.

### Relationship of serum IS levels with renal function and age

**Figure 1:**
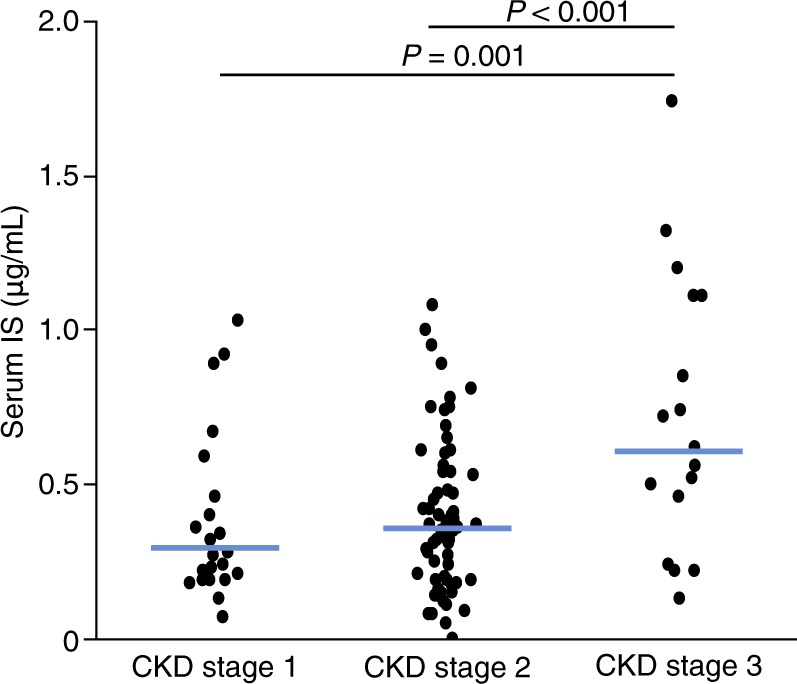
Serum levels of IS are increased in patients with reduced renal function. CKD, chronic kidney disease; IS, indoxyl sulphate.

**Figure 2:**
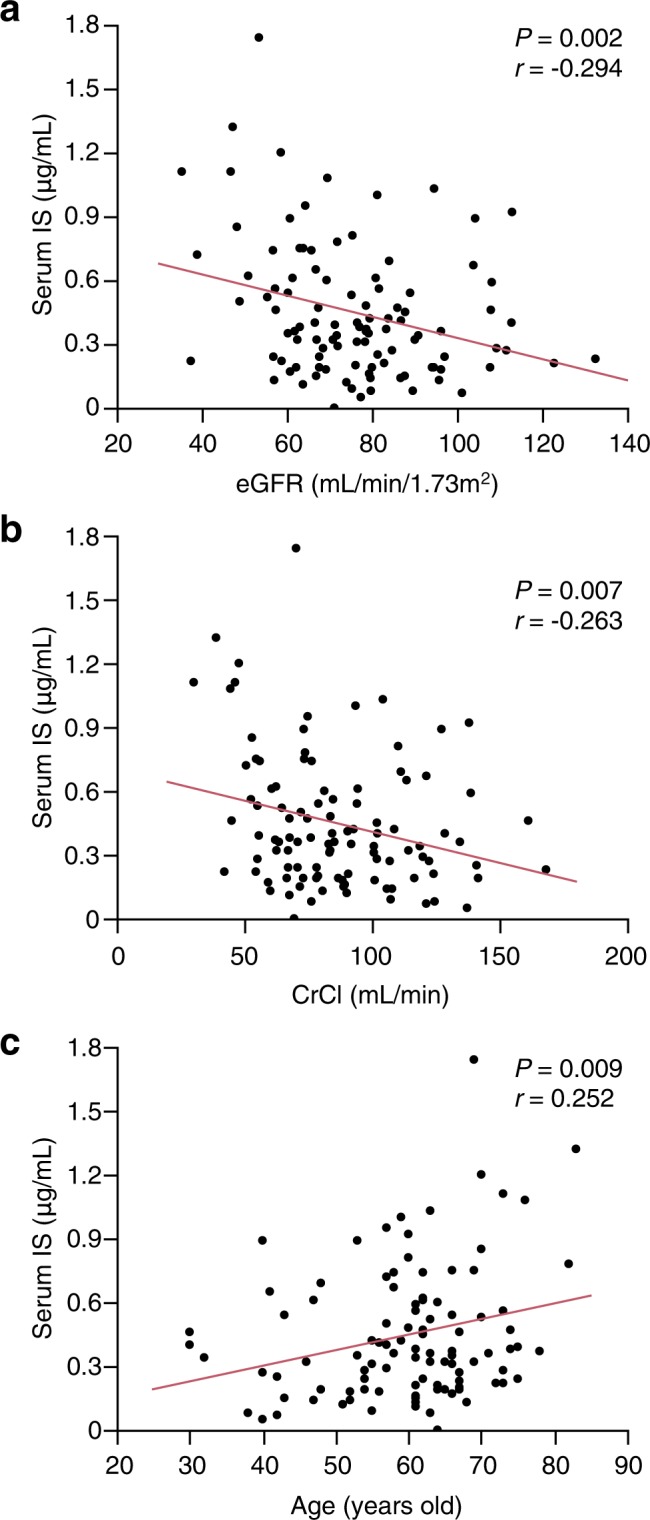
Correlations between serum levels of IS and eGFR (**a**), CrCl (**b**), or age (**c**). CrCl, creatinine clearance; eGFR, estimated glomerular filtration rate; IS, indoxyl sulphate.

Serum IS levels were significantly increased in patients with CKD stage 3 (Fig. 1). However, the correlations of serum IS levels with eGFR (*r* = −0.295, *P* = 0.002) and CrCl (*r* = −0.263, *P* = 0.007) were weak (Fig. 2a,b). IS levels were also weakly correlated with age (*r* = 0.253, *P* = 0.009; Fig. 2c).

IAA is also a uremic toxin and belongs to the family of indolic uremic solutes, such as IS. Serum levels of IAA were positively correlated with those of IS (*r* = 0.443, *P* < 0.001; Supplementary Fig. S1). In this cohort, unlike IS, IAA levels did not differ statistically significantly among patients with CKD stage 1, 2, and 3 (Supplementary Fig. S2). IAA levels were weakly correlated with eGFR (*r* = −0.255, *P* = 0.009), but not with CrCl (r = −0.102, *P* = 0.30) or age (*r* = −0.029, *P* = 0.77) (Supplementary Fig. S3).

### Demographic and clinical characteristics of patients with high IS levels

Among the 105 patients in our study group, 23 patients (21.9%) were categorized into the high IS (≥0.65 μg/mL) and 82 patients (78.1%) into the low IS group (<0.65 μg/mL; Table 1) according to the optimal cut-off value. There were no significant differences in sex or age between the two groups. The duration after diagnosis of AF was significantly longer in the high IS group (*P* = 0.010). Patients with high IS levels had lower eGFR (*P* = 0.019), an increased LAVI (*P* = 0.012), an elevated BNP (*P* = 0.042), and were more likely to have been treated with beta-blockers before ablation (*P* = 0.003). After ablation, the proportion of patients taking class III anti-arrhythmic drugs (AAD) was significantly higher in the high-IS group than in the low-IS group (*P* = 0.004, Supplementary Table S1).

### AF-free survival

**Figure 3:**
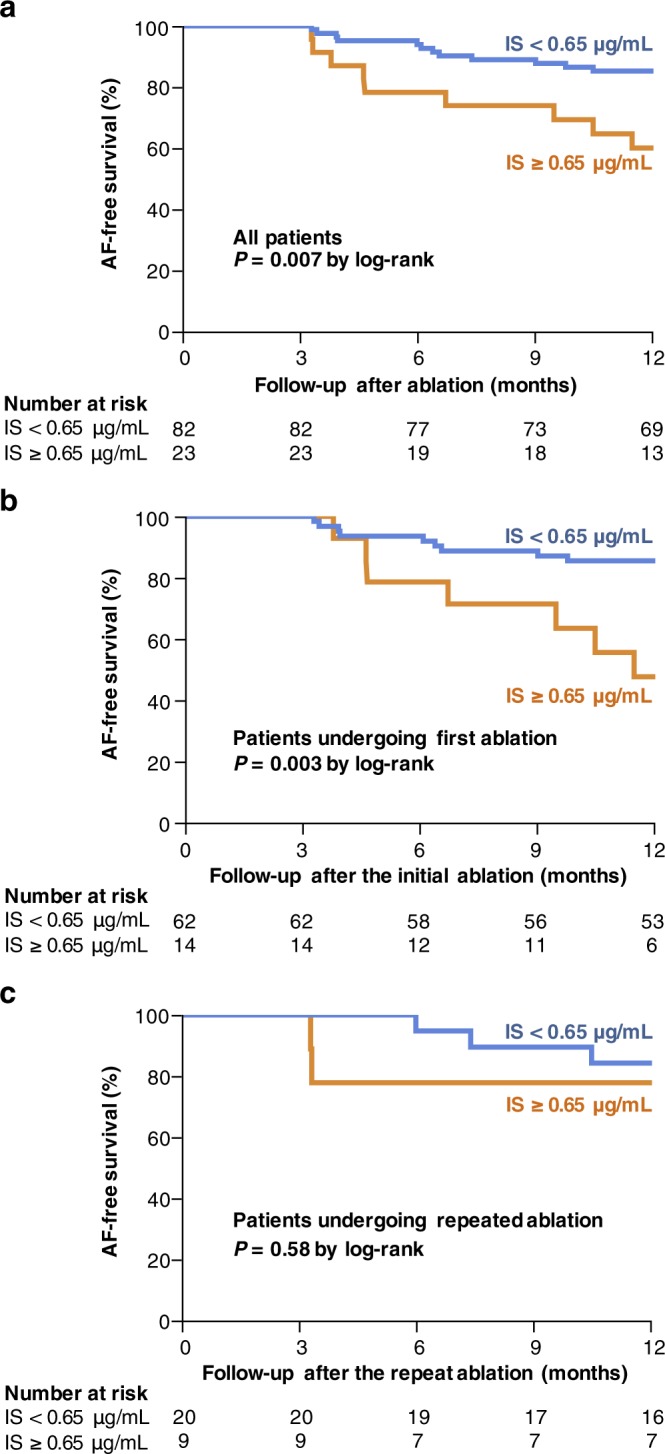
Impact of IS levels on the recurrence of AF after catheter ablation. The AF-free survival rates are shown for the whole cohort (**a**), patients undergoing a first AF ablation (**b**), and patients undergoing a repeat AF ablation (**c**) according to IS levels. The numbers at the bottom of the graph indicates the number of ‘at risk’ patients in each follow-up month. AF, atrial fibrillation; IS, indoxyl sulphate.

The 1-year AF-free survival was significantly lower in patients with a high serum IS level than in those with a low IS level (60.1 ± 10.4% *vs* 85.2 ± 3.9%, *P* = 0.007; Fig. 3a). However, the 1-year AF-free survival did not differ (*P* = 0.25) between patients with high IAA levels (85.9 ± 5.8%) and those with low IAA levels (76.6 ± 5.1%) (Supplementary Fig. S4).

Overall, in our study group, 76 patients (72.4%) underwent a first AF ablation, with the remaining 29 (27.6%) undergoing a repeat AF ablation. Among the 29 patients with recurrent AF, recovery of electrical conduction between the PVs and the LA was identified in 26 patients (89.7%). Among patients who underwent a first AF ablation, the 1-year estimates of AF-free survival were 47.6 ± 13.8% for those with a high serum IS level and 85.5 ± 4.5% for those with a low IS level (*P* = 0.003; Fig. 3b). In contrast, among patients who underwent a repeat AF ablation (Fig. 3c), the 1-year AF-free survival did not significantly differ (*P* = 0.58) between patients with high IS levels (77.9 ± 13.9%) and those with low IS levels (84.2 ± 8.4%).

### Predictive value of IS in AF recurrence

On univariate analysis, an IS level ≥ 0.65 μg/mL was associated with an increased risk of AF recurrence (hazard ratio [HR]: 3.10, 95% CI: 1.26–7.32; *P* = 0.015; Table 3). After adjustment for age and sex, an IS level ≥ 0.65 μg/mL remained associated with increased risk of AF recurrence (HR: 3.76, 95% CI: 1.52–9.03; *P* = 0.005; Table 3). In the multivariate Cox proportional hazard model, adjusted for age, sex, duration after AF diagnosis, beta-blocker use before ablation, LAVI, BNP, Class III AAD use after ablation, as well as eGFR or CKD stage, an IS level ≥ 0.65 μg/mL remained as an independent predictor of AF recurrence (HR 3.60, 95% CI: 1.12–11.0, *P* = 0.032; and HR 3.67, 95% CI: 1.13–11.7, *P* = 0.031, respectively; Table 3).

**Table 3:** Impact of a level of indoxyl sulphate ≥ 0.65 μg/mL on atrial fibrillation recurrence.

	HR	95% CI	*P-*value
Unadjusted analysis	3.10	1.26−7.32	0.015
**Analysis adjusted for:**			
Age and sex	3.76	1.52−9.03	0.005
Age, sex, duration after diagnosis of AF, beta-blocker use before ablation, LAVI, BNP, Class III AAD use after ablation, and eGFR	3.60	1.12−11.0	0.032
Age, sex, duration after diagnosis of AF, beta-blocker use before ablation, LAVI, BNP, Class III AAD use after ablation, and CKD stage	3.67	1.13−11.7	0.031

AAD, anti-arrhythmic drug; AF, atrial fibrillation; BNP, B-type natriuretic peptide; CI, confidence interval; CKD, chronic kidney disease; eGFR, estimated glomerular filtration rate; HR, hazard ratio; LAVI, left atrial volume index.

## Discussion

In this prospective observational study, we identified high IS levels as a strong and independent predictor of AF recurrence in patients undergoing successful catheter ablation. To the best of our knowledge, this association of uremic toxins with the outcomes of AF ablation has not been described previously.

The high AF recurrence rate after ablation in patients with CKD is indicative of a possible involvement of uremic toxins in AF pathogenesis. However, no clinical studies to date have demonstrated the involvement of uremic toxins in AF recurrence. Here, we showed that patients with a higher IS levels exhibited a higher rate of AF recurrence after ablation, with serum IS being a significant predictor of AF recurrence, even after adjustment for eGFR or the stage of CKD. Thus, it seems that IS has a predictive role in AF recurrence.

In recent years, increasing attention has been paid to the relationship between IS and cardiovascular diseases among patients with CKD. Barreto *et al*.^20^ reported that a higher serum level of IS was associated with an increased overall mortality and cardiovascular-specific mortality among patients with CKD. Furthermore, Lin *et al*.^21^ indicated that IS was a valuable marker in predicting cardiovascular events in patients with advanced CKD. Other researchers have reported association of a high IS level with increased risk of LV diastolic dysfunction^22,23^.

In our study cohort, the IS concentration correlated poorly with eGFR (Fig. 1), which was consistent with the findings from other groups^24,25^. IS is a gut-derived uremic toxin and factors other than the GFR, such as renal tubular secretion, diet and intestinal absorption, and gut microbiota metabolism, may affect IS concentration^26^.

Recent animal studies have revealed the causative role of IS in promoting AF remodelling. Chen *et al*.^15^ reported that IS induced the occurrence of delayed after-depolarizations and burst firing in PVs isolated from rabbits. They also showed that IS-treated PV cardiomyocytes had a larger Ca^2+^ leak than control PV cardiomyocytes^15^. Aoki *et al*.^16^ showed that, in rat models of renal failure induced by 5/6 nephrectomy, administration of AST-120, which is commonly used in clinical practice as an absorbent of uremic toxins, attenuated oxidative stress, inflammation, and fibrosis in the LA, and decreased AF inducibility *in vivo*. In cultured atrial fibroblasts, incubation with IS upregulated the expression of oxidative stress markers NOX2/NOX4 and malondialdehyde, along with an increase in inflammatory and profibrotic signalling molecules, such as monocyte chemoattractant protein 1 (MCP-1), transforming growth factor β1 (TGF-β1), α-smooth muscle actin (α-SMA), and collagen I^16^. These results indicate a direct effect of IS on the progression of the AF substrate. Therefore, IS may become a novel therapeutic target for AF.

The prevalence of AF among a large population of patients with CKD was found to be 2- to 3-fold higher than that in the general population^27,28^. Moreover, the prevalence of AF among dialysis patients has been reported to range between 7% and 27%^29–32^, as compared to 0.4% to 1.0% in the general population^33^, indicating that haemodialysis may not eliminate the risk of AF in CKD. Therefore, non-dialyzable uremic factors were considered responsible for AF-promoting remodelling. IS is a poorly dialyzable uremic toxin due to its high protein binding. Even after haemodialysis, the serum IS levels still remain high^34^. Therefore, accumulating IS may directly contribute to atrial remodelling in patients with CKD.

### Limitations

The study had some limitations. First, compared to patients in the low IS level group, the duration after AF diagnosis had been longer in the cohort of patients with high IS levels; this group also had a greater prevalence of beta-blocker use, increased LAVI, and worse renal function, which would be sources of possible residual confounding factors. Furthermore, our study evaluated a heterogeneous population, including patients with paroxysmal as well as persistent AF. Because of the small sample size, no separate analysis was possible between these different groups. Consequently, we cannot rule out different biomarker patterns between these two entities of AF. Second, the diagnosis of AF recurrence was based on the occurrence of symptoms and periodic and occasional ECG recordings and Holter ECG findings. Therefore, some patients with asymptomatic AF recurrence might have been missed. Third, our cohort included individuals who underwent AF ablation at our institution between 2009 and 2011. During that period, advanced technologies that are widely used today, including the contact force-sensing catheter and balloon ablation, were not approved for use in Japan. These newer technologies have been reported to increase the durability of PV isolation and reduce AF recurrence^35,36^. Further studies may be needed to determine whether high IS levels can predict AF recurrence in the current setting of AF ablation. Fourth, patients who had haemodynamically significant valvular heart disease or stage 4 or 5 CKD, or received dialysis were excluded from this study. Further studies are needed to determine whether high IS levels can also predict AF recurrence in those patients. Fifth, it has been reported that there were some diurnal and inter-day variations in the circulating IS concentrations^37^, and dietary protein intake affected the IS concentrations^38^. However, in this study, blood samples were collected at a single time point, but were not collected at the same time of the day for all patients, and were not standardized with respect to food intake. Finally, this study was performed in a single centre with a relatively small sample size and a short follow-up period. Therefore, further studies with more patients and a longer follow-up period may be needed to confirm our study results.

## Conclusion

Serum IS levels are associated with recurrence of AF after catheter ablation. Therefore, the IS level should be considered in the prediction of recurrence after ablation.

## Electronic supplementary material

Supplementary Information

## Data Availability

The datasets generated and/or analysed during the current study are available from the corresponding author upon reasonable request.
