# Supplementary material for: Indoxyl Sulphate is Associated with Atrial Fibrillation Recurrence after Catheter Ablation
Source: Sci Rep. 2018 Nov 22;8:17276. doi: 10.1038/s41598-018-35226-5 (PMC6250674; doi:10.1038/s41598-018-35226-5)
Supplement: Supplementary file 1 — Supplementary Information [file 41598_2018_35226_MOESM1_ESM.pdf]

Indoxyl Sulfate is Associated with Atrial Fibrillation Recurrence after Catheter Ablation

Fumi Yamagami, Kazuko Tajiri, Kosuke Doki, Masayuki Hattori, Jyunya Honda, Satoshi Aita, Tomohiko Harunari, Hiro Yamasaki, Nobuyuki Murakoshi, Yukio Sekiguchi, Masato Homma, Naohiko Takahashi, Kazutaka Aonuma, Akihiko Nogami, Masaki Ieda

Supplementary Table

Supplementary Table 1. Anti-arrhythmic medications after catheter ablation for AF

	All (n = 105)	IS ≥ 0.65 μg/mL (n = 23)	IS < 0.65 μg/mL (n = 82)	*P*-value
Class I AADs	27 (25.7)	6 (26.1)	21 (25.6)	0.96
Class III AADs	25 (23.8)	11 (47.8)	14 (17.1)	0.004
Class IV AADs	9 (8.6)	4 (17.4)	5 (6.1)	0.11

AAD; anti-arrhythmic drug; AF, atrial fibrillation; IS, indoxyl sulfate

Supplementary Figures

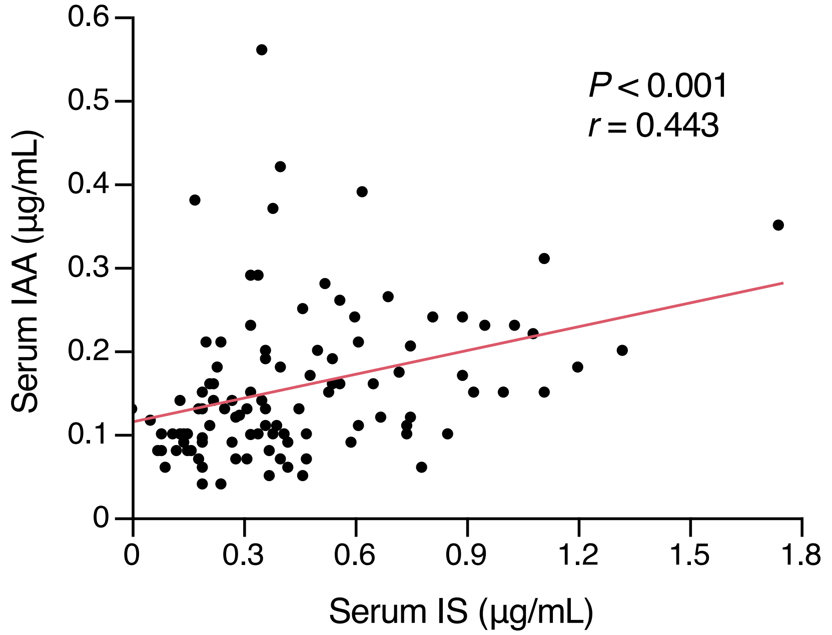

Supplementary Fig. S1. Relationship between serum levels of IS and IAA. IAA = indole-3 acetic acid, IS = indoxyl sulfate.

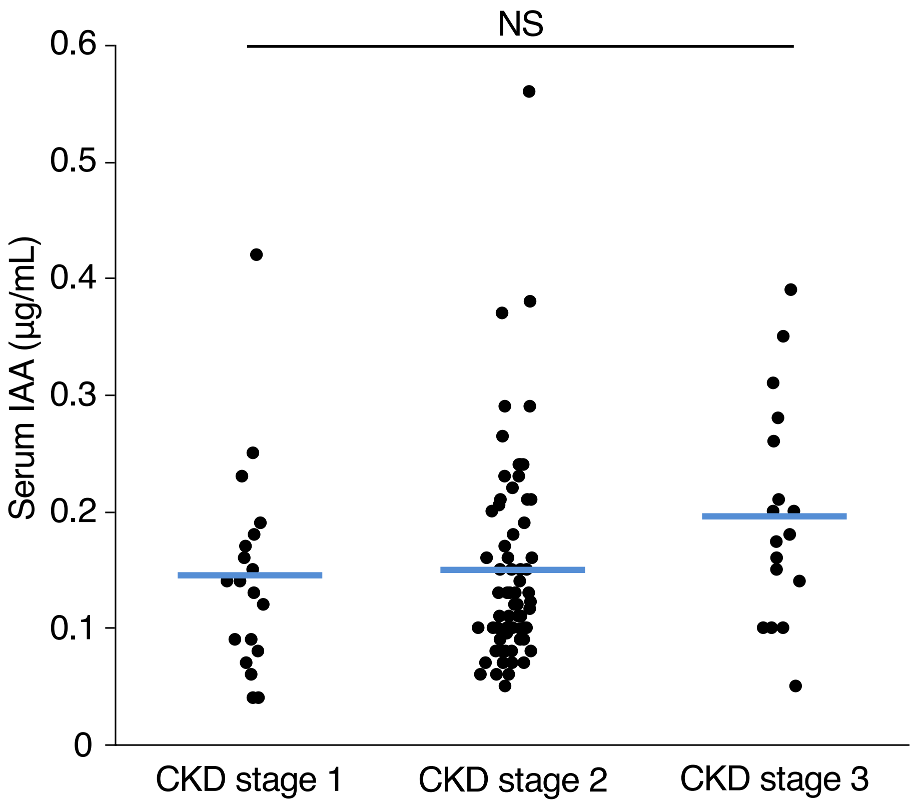

Supplementary Fig. S2. Serum levels of IAA and CKD stages. CKD = chronic kidney disease, IAA = indole-3 acetic acid.

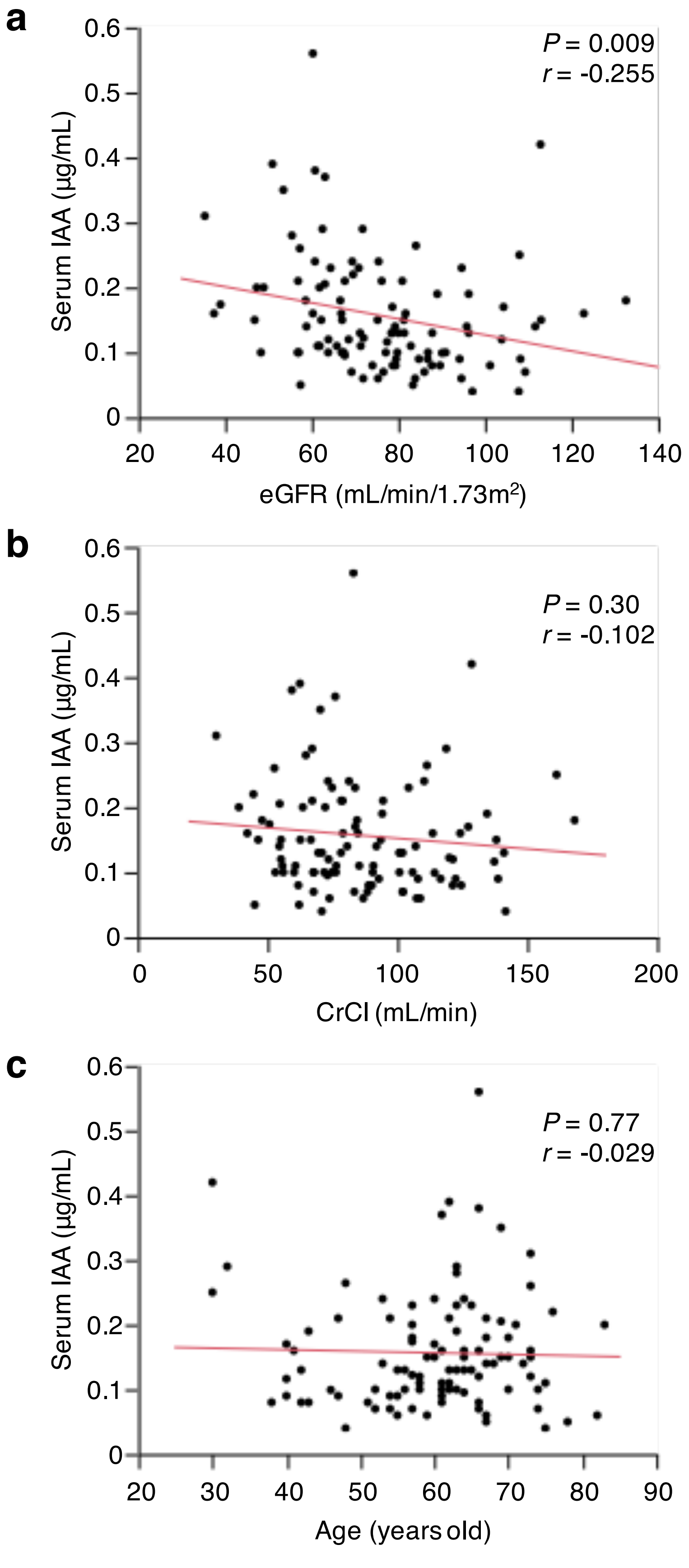

Supplementary Fig. S3. Correlations between serum levels of IAA and eGFR (a), CrCl (b), or age (c). CrCl = creatinine clearance, eGFR = estimated glomerular filtration rate, IAA = indole-3 acetic acid.

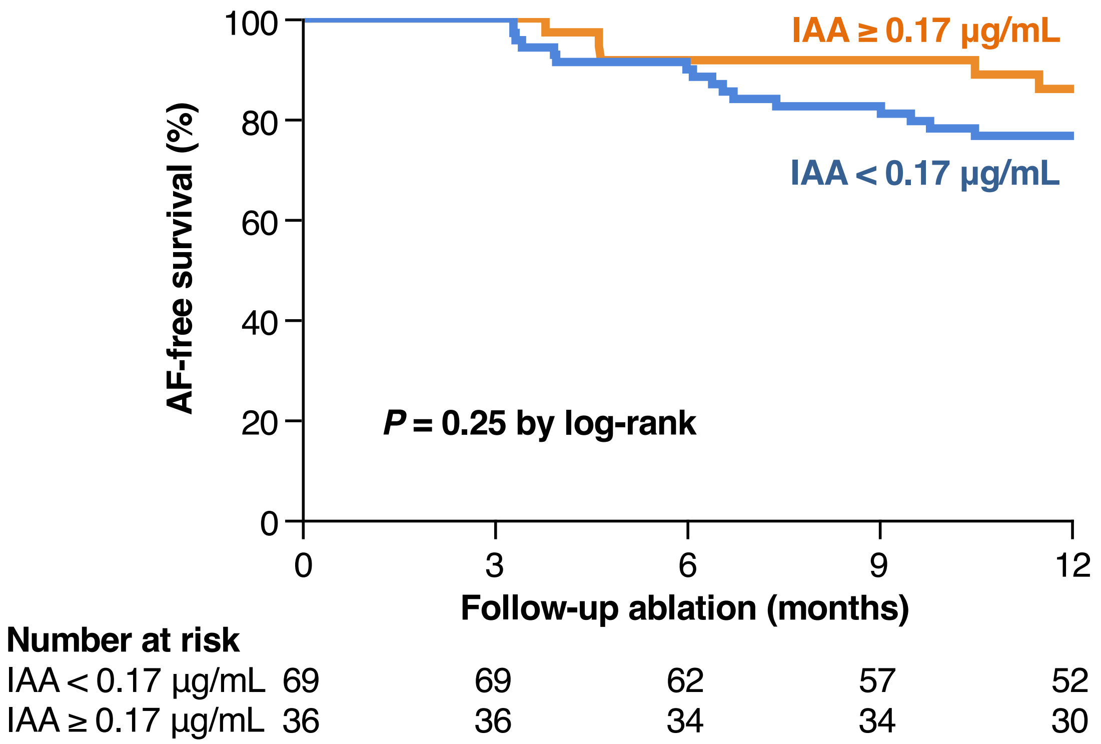

Supplementary Fig. S4. Impact of IAA levels on the recurrence of AF after catheter ablation. The numbers at the bottom of the graph indicates the number of 'at risk' patients at each follow-up month. AF = atrial fibrillation, IAA = indole-3 acetic acid.
